# The Association Between Meningioma and Breast Cancer: A Systematic Review and Meta-analysis

**DOI:** 10.1001/jamanetworkopen.2023.18620

**Published:** 2023-06-16

**Authors:** Aurélie Degeneffe, Viviane De Maertelaer, Olivier De Witte, Florence Lefranc

**Affiliations:** 1 Department of Neurosurgery, Hôpital Universitaire de Bruxelles, Université Libre de Bruxelles, Brussels, Belgium; 2 Biostatistical Unit, Institute of Interdisciplinary Research in Human and Molecular Biology, Faculty of Medicine, Université Libre de Bruxelles, Brussels, Belgium

## Abstract

**Importance:**

A potential relationship between meningioma and breast cancer was suggested 70 years ago. However, to date, no conclusive evidence is available on this topic.

**Objective:**

To provide a comprehensive review of the literature on the association of meningioma with breast cancer, supported by a meta-analysis.

**Data Sources:**

A systematic PubMed search was performed up to April 2023 to identify articles on the association of meningioma with breast cancer. The following key words were used strategically: *meningioma*, *breast cancer*, *breast carcinoma*, *association*, *relation*.

**Study Selection:**

All studies reporting women diagnosed with meningioma and breast cancer were identified. The search strategy was not limited by study design or publication date but only included articles in English. Additional articles were identified via citation searching. Studies reporting a complete population of meningiomas or breast cancer patients throughout a specific study period and a proportion of patients with a second pathology could be used for the meta-analysis.

**Data Extraction and Synthesis:**

Data extraction was performed by 2 authors in accordance with the Preferred Reporting Items for Systematic Reviews (PRISMA) statement. Meta-analyses regarding both populations were performed using a random-effects model. Risk of bias was assessed.

**Main Outcomes and Measures:**

The main measures were whether there was an increased prevalence of breast cancer in female patients with meningioma and whether there was an increased prevalence of meningioma in female patients with breast cancer.

**Results:**

A total of 51 retrospective studies (case reports, case series, and cancer registry reports) describing 2238 patients with both diseases were identified; 18 studies qualified for prevalence analyses and meta-analysis. The random-effects meta-analysis (13 studies) revealed a significantly greater prevalence of breast cancer in female patients with meningioma than in the overall population (odds ratio [OR], 9.87; 95% CI, 7.31-13.32). Meningioma incidence in patients with breast cancer (11 studies) was greater than that in the baseline population; however, the difference according to the random-effects model was not statistically significant (OR, 1.41; 95% CI, 0.99-2.02).

**Conclusions and Relevance:**

This large systematic review and the meta-analysis on the association between meningioma and breast cancer found nearly 10-fold higher odds of breast cancer in female patients with meningioma compared with the general female population. These findings suggest that female patients with meningioma should be screened more intensively for breast cancer. Further research is required to identify the factors causing this association.

## Key Points

**Question:**

Is there a lifetime higher risk of meningioma among female patients with breast cancer or of breast cancer among female patients with meningioma compared with the overall female population?

**Findings:**

This systematic review and meta-analysis of 51 studies describing 2238 patients found that female patients diagnosed with meningioma were at significantly higher risk of breast cancer compared with the general population. The odds ratio of meningioma in female patients with breast cancer was not statistically significant.

**Meaning:**

These findings suggest guidelines should be updated regarding screening for these 2 diseases in female patients, specifically regarding breast cancer in female patients with meningioma.

## Teaser

This systematic review and meta-analysis describes the literature on the associations between meningioma and breast cancer and analyzes the available data.

## Introduction

The most common adult primary intracranial neoplasms are meningiomas arising from the meningothelial cells on the outer surface of the arachnoid villi.^1,2^ The age-adjusted incidence of meningiomas reported in the United States is 12.41 per 100 000 female individuals per year (2014-2018), and it continues to increase, according to the Central Brain Tumor Registry United States.^1^ The peak incidence of this typically benign tumor (ie, benign in 98% of patients; World Health Organization grade one, 80% of patients; grade two, 18.3% of patients; and grade three, 1.6% of patients) occurs from ages 50 to 70 years.^3,4^ In addition, this is a tumor with a clear female predisposition, occurring with a female to male ratio of 2.3 to 1.^1,4^

Another typical female disease is breast cancer. With its continuously increasing incidence, this is the second most common type of cancer in female patients.^5^ Its worldwide incidence was 47.8 per 100 000 female individuals in 2020, according to the Global Cancer Observatory.^5^

Seven decades ago,^6^ a potential association between meningioma and breast cancer was first described. Since the initial case report by Lapresle et al^6^ in 1952 describing an individual with both illnesses, multiple case reports and a few larger patient series have suggested an association between meningioma and breast cancer. Schoenberg et al^7^ established this unique association in 1975, reporting 8 patients with breast cancer in a cancer registry of 402 patients with meningioma instead of the 3.4 expected patients.^7^

More recently, studies have been conducted on a larger scale using cancer registries. However, these results demonstrate divergent conclusions. A 2020 study by Lopez-Rivera et al^8^ found an increased risk of meningioma after a breast cancer diagnosis.^8^ A 2016 study by Ji et al^9^ found a similar outcome in a portion of their investigated cohort. However, in 2013, Criscittiello et al^10^ reported no higher risk of subsequent meningioma in female patients with breast cancer. As a result, meningioma and breast cancer have not yet been conclusively linked.

Therefore, the aim of our study is to provide a comprehensive review of the published literature related to the association between meningioma and breast cancer, supported by prevalence analyses and a meta-analysis. We will present results considering both populations, specifically regarding the prevalence of meningioma among individuals with breast cancer, as well as the prevalence of breast cancer among individuals with meningioma.

## Methods

### Literature Search Strategy

This systematic review was performed up to April 2023 in accordance with the Preferred Reporting Items for Systematic Reviews and Meta-analyses (PRISMA) reporting guideline. All articles describing patients diagnosed with both intracranial or spinal meningioma and breast cancer or describing breast cancer and intracranial collision tumors were considered for review. Article selection was performed by a systematic search of PubMed to identify articles regarding an association between meningioma and breast cancer. The following key words were used: *meningioma* AND *breast cancer* OR *breast carcinoma* AND *association* OR *relation*. The search strategy was not limited by study design or publication date but only included key words in the English language; thus, at least the abstract had to be in English. Additional articles were identified via citation searching. The titles and abstracts of all records were screened by A.D. and V.D.M. Data regarding the first diagnosed tumor, histology, hormonal receptor expression, treatment received, age at diagnosis of the first tumor, and time between diagnoses of the 2 diseases were collected from full texts when available. Data extraction was performed by A.D. and V.D.M.

### Selection Criteria

Subsequently, a selection was made of articles for which odds ratio (OR) calculations and prevalence analysis could be carried out on the basis of the following inclusion and exclusion criteria: studies were only included in the analysis if a complete population of patients with meningioma or breast cancer was presented throughout a specific study period, together with a proportion of patients with a second condition, whether breast cancer or meningioma. Consequently, case reports and larger case series lacking a total population were excluded. Studies with overlapping data from the same cancer registry were excluded.

### Statistical Analysis

#### Prevalence Analysis

After selecting articles suitable for the analysis, articles were classified into 2 groups. The first group consisted of studies starting from a population of female patients with meningioma in which the number of patients who had breast cancer also was examined. Breast cancer may have been diagnosed before or after meningioma. The second group consisted of studies starting with female patients with breast cancer and involved examining the number of female patients with meningiomas regardless of whether the diagnosis of meningioma was made before or after the diagnosis of breast cancer during the study period. Studies performing bidirectional analyses were classified into each of these 2 groups. Few studies indicated which diagnosis was first. Due to a lack of data regarding the first diagnosed tumor, histology, hormonal receptor expression, treatment received, age at diagnosis of the first tumor, and time between diagnoses of the 2 diseases in most of the literature reviewed, no subanalyses could be conducted.

The proportions of patients with the second condition were calculated for each study. For group 1, the proportion of patients with breast cancer per study (prevalence per study) in the meningioma group was determined. This was then compared with the 2020 breast cancer prevalence, adjusted for the country where the study was conducted. Using MedCalc version 20.111 (MedCalc Software), a 1-proportion test was run to determine whether the population under study deviated significantly from the general prevalence. The proportion of patients with meningioma per study (prevalence per study) in the breast cancer group was determined. This was compared with the 2020 meningioma prevalence in Belgium. Identical procedures were followed for group 2.

#### Meta-analysis

Based on these results, a meta-analysis was carried out, gathering results using MedCalc, SPSS Statistics version 28 (IBM), and NCSS version 10 (NCSS Statistical Software). Heterogeneity between studies was evaluated using Cochran *Q* statistics and the *I*^2^ statistic. A significant *Q* statistic value suggests that the homogeneity hypothesis of the association set can be rejected, while an *I*^2^ value greater than 50% indicates strong heterogeneity. A restricted maximum likelihood (REML) random-effects model was used. ORs were considered. Egger and Peter tests were used to detect possible publication bias. Meta-analyses were conducted for each of the 2 groups. The Meta-analyses of Observational Studies in Epidemiology (MOOSE) checklist was used as a guide.

## Results

### Systematic Review

**Figure 1.**
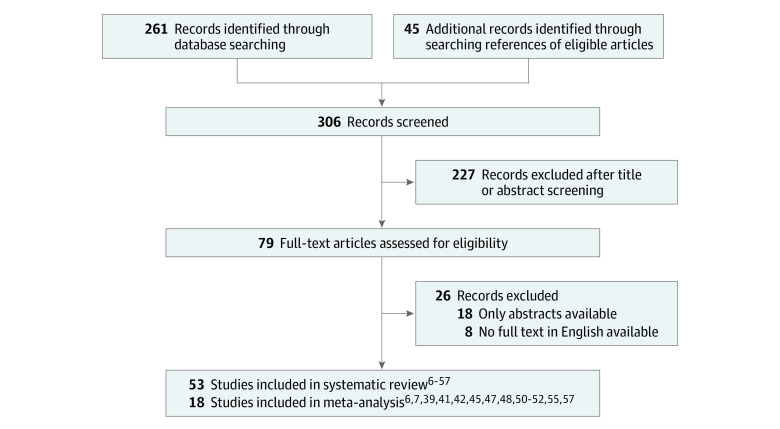
Flowchart for Study Selection

Figure 1 shows the flowchart for the study selection process at different stages. A total of 306 articles were identified: 261 records were found by searching the PubMed database, and 45 studies were found manually and cross-referenced with identified articles’ corresponding reference lists. English-language full text was not available for 26 records; hence, they were excluded. Finally, 53 studies^6,7,8,9,10,11,12,13,14,15,16,17,18,19,20,21,22,23,24,25,26,27,28,29,30,31,32,33,34,35,36,37,38,39,40,41,42,43,44,45,46,47,48,49,50,51,52,53,54,55,56,57^ were included for this systematic review: 27 case reports,^6,11,12,13,14,15,16,17,18,19,20,21,22,23,24,25,26,27,28,29,30,31,32,33,34,35,36^ 14 case series^10,37,38,39,40,41,42,43,44,45,46,47,48^ and 12 cancer registry studies.^7,8,9,49,50,51,52,53,54,55,56,57^ The case reports, case series, and cancer registry studies described a total of 2238 individual patients diagnosed with both pathologies, including patients with intracranial collision tumors.

### Study Selection

Twenty-three articles were selected as potentially suitable for the analysis of odds, ORs, and prevalence. After the exclusion of 5 studies^43,49,53,54,56^ for the reasons described in the Table, 18 studies^6,7,8,9,10,37,39,41,42,44,45,47,48,50,51,52,55,57^ were ultimately included for these analyses. The study by Maiuri et al^43^ found a particularly low value for the breast cancer OR.^43^ We observed an underreporting bias in this instance. Of 660 patients with meningioma who underwent surgery, 3 had breast cancer and brain metastases. Maiuri et al^43^ focused solely on the number of patients with breast cancer who had brain metastases. Patients with nonmetastatic breast cancer were not discussed in the study by Maiuri et al^43^; therefore, this study was excluded from the subsequent analyses.

**Table.** List of Excluded Articles

Publication	Reason for exclusion
Adami et al,^49^ 1984	Overlap of data with Ji et al,^9^ 2016
Jacobs et al,^53^ 1992	Overlap of data with Jacobs et al,^52^ 1987
Malmer et al,^56^ 2000	Overlap of data with Ji et al,^9^ 2016
Maiuri et al,^43^ 2002	Reporting bias
Milano et al,^54^ 2014	Overlap of data with Lopez-Rivera et al,^8^ 2020

Eight studies^6,7,41,42,45,48,52,57^ analyzed a population of patients with meningioma, 5 studies^8,9,10,37,44^ analyzed a population of patients with breast cancer, and the other 5 studies^39,47,50,51,55^ conducted a bidirectional analysis. The study by Ji et al^9^ separated analyses into 2 sections, distinguishing between a cohort of patients diagnosed with breast cancer prior to 1987 and a cohort diagnosed after 1987. The latter group was characterized as having been exposed to tamoxifen. Consequently, these 2 components of the study were regarded as independent units in the analysis.

This yielded a total of 13 analyses based on the meningioma population and 11 analyses based on the breast cancer population. As only retrospective studies and case series were available, the level of evidence of the included studies was 3 or 4, according to the Oxford Centre for Evidence-based Medicine for ratings of individual studies.

Using the incidence ratios per year, the number of observed cases can be compared with the number of expected cases, and thus, the standardized incidence ratio (SIR) can be calculated. Of 13 studies^6,7,39,41,42,45,47,48,50,51,52,55,57^ assessed for the meningioma population, 6 expressed the risk of developing breast cancer as SIR or similar ratios. The most recent study, conducted by Ben Lassan et al,^57^ reported an SIR of 1.6 (95% CI, 1.4-1.9) for invasive breast cancer and 1.7 (95% CI, 1-2.6) for in situ breast cancer in their cohort of female individuals diagnosed with meningioma. A study by Rao et al^55^ reported that the cumulative observed rate of breast cancer in female individuals previously diagnosed with meningioma was 58 times the expected rate.^55^ A study by Custer et al^50^ found that the risk of breast cancer was not significantly increased after a diagnosis of meningioma in their cohort (SIR, 1.54; 95% CI, 0.77-2.75).^50^ A study by Helseth et al^51^ identified a ratio of observed vs expected breast cancer of 21 to 11.9 (SIR, 1.75; 95% CI, 1.08-2.68) with regard to developing a subsequent breast cancer.^51^ Two studies by Jacobs et al^52,53^ reported an SIR of 1.43 (95% CI, 0.74-2.50), regardless of whether the breast cancer diagnosis manifested before or after the meningioma diagnosis. Last, the 1975 study by Schoenberg et al^7^ was the first to identify a statistically significantly increased risk of breast cancer (SIR, 2.37) in the presence of meningiomas in female patients.^7^

Of 11 studies^8,9,10,37,39,44,47,50,51,55^ assessed for the breast cancer population, 6 expressed the risk of developing meningioma as SIR or similar. The study by Helseth et al^51^ reported an SIR of 1.54 (95% CI, 0.97-2.34), Custer et al^50^ reported an SIR of 1.40 (95% CI, 0.67-2.58), Rao et al^55^ found 80 (95% CI, 72-89), Ji et al^9^ reported an SIR of 1.53 (95% CI, 1.30-1.81) without tamoxifen exposure and 1.06 (95% CI, 0.84-1.32) with tamoxifen exposure, and Lopez-Rivera et al^8^ discovered an SIR of 1.26 (95% CI, 1.19-1.33) within the first 5 years after the diagnosis of breast cancer. According to Lopez-Rivera et al,^8^ patients with breast cancer aged 18 to 49 years (SIR, 2.16; 95% CI, 1.78-2.61) and those with a more advanced tumor stage (stage IV: SIR, 2.39; 95% CI, 1.71-3.25; *P* = .05) were at a higher risk of developing meningioma. Patients in subgroups according to hormone receptor expression and treatment modality were at comparable risk vs the overall population.^8^ A study by Criscitiello et al^10^ found no statistically significant increased risk of developing meningioma.

### Meta-analysis

#### Meningioma Population

**Figure 2.**
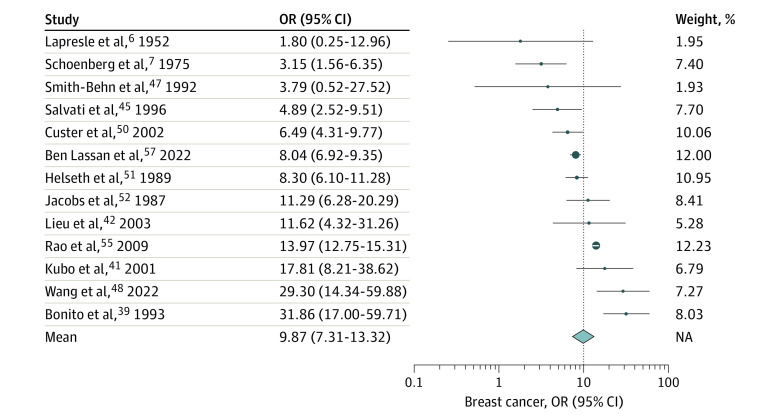
Estimated Odds Ratios (ORs) for Breast Cancer in Each Meningioma Study and Overall Dots indicate estimates; size of dots, weight; whiskers, 95% CIs; diamond, overall association estimated using a random-effects model; NA, not applicable.

When comparing the studies,^6,7,39,41,42,45,47,48,50,51,52,55,57^ the heterogeneity test results were highly statistically significant (Cochran *Q* = 92.9; *df* = 12; *P* < .001) with an *I*^2^ of 93%, indicating that the between-study heterogeneity was high. Therefore, a random-effects model was chosen for the meta-analysis. Possible publication bias was detected (Egger linear regression intercept [SE], 2.57 [0.36]; *P* < .001; Peter linear regression intercept [SE], 2.12 [0.24] *P* < .001). The meta-analysis, based on the random-effects model, revealed a considerably greater prevalence of breast cancer in female patients with meningioma compared with the baseline prevalence of breast cancer in the general population (OR, 9.87; 95% CI, 7.31-13.32). Figure 2 illustrates the results of the meta-analysis.

#### Breast Cancer Population

**Figure 3.**
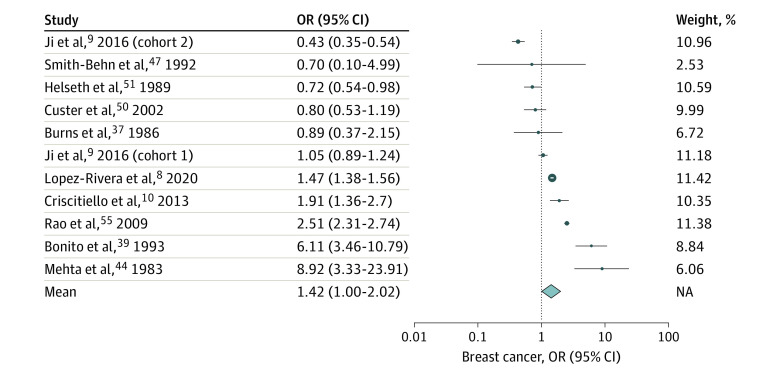
Estimated Odds Ratios (ORs) for Meningioma in Each Breast Cancer Study and Overall The study by Ji et al^9^ separated analyses into 2 sections, distinguishing between a cohort of patients diagnosed with breast cancer prior to 1987 (cohort 1) and a cohort diagnosed after 1987 (cohort 2). The study by Mehta et al^44^ reported on an analysis of an autopsy-based retrospective study of patients with end-stage breast cancer, conducted by J. B. Posner in 1982. Dots indicate estimates; size of dots, weight; whiskers, 95% CIs; diamond, overall association estimated using a random-effects model; NA, not applicable.

When comparing the breast cancer studies,^8,9,10,37,39,44,47,50,51,55^ the heterogeneity test results were highly statistically significant (Cochran *Q* = 345.5; *df* = 10; *P* < .001), with an *I*^2^ of 98%, indicating that the between-study heterogeneity was high. Therefore, a random-effects model was chosen for the meta-analysis. No publication bias was observed using Egger test (linear regression intercept [SE], 0.25 [0.40]; *P* = .56) nor Peter test (linear regression intercept [SE], 0.38 [0.28]; *P* = .22). The random-effects model did not reach statistical significance (OR, 1.41; 95% CI, 0.99-2.02). Figure 3 illustrates the results of the meta-analysis.

## Discussion

To our knowledge, this systematic review and meta-analysis is both the largest systematic review and the first-meta-analysis that has been conducted on the association between meningioma and breast cancer. The main objective of the study was to clarify whether there was an increased prevalence of breast cancer among female patients with meningioma, and vice versa. Based on the meta-analysis of 18 retrospective studies,^6,7,8,9,10,37,39,41,42,44,45,47,48,50,51,52,55,57^ there was an association between these diseases.

In female patients with meningioma, we found approximately 10-fold higher odds of developing breast cancer during their lifetime based on 13 cohorts.^6,7,39,41,42,45,47,48,50,51,52,55,57^ In previous literature, the risk of having breast cancer while diagnosed with a meningioma was explored using state and national cancer registries. To our knowledge, only 3 cohorts have previously reported a statistically significant increase in the risk of developing breast cancer in female patients with meningiomas.^7,51,55^

In female patients with breast cancer, we found that odds of developing meningioma during the lifetime were increased by 1.42-fold, based on 11 cohorts. There was no statistically significantly increased prevalence of meningioma in the population of female patients with breast cancer compared with the control population. In 5 studies,^8,9,50,51,55^ an SIR for developing a subsequent meningioma after breast cancer diagnosis in females was reported. Only 3 previous cohorts^8,9,55^ reported a statistically significant increase in the likelihood of developing meningioma in female patients with breast cancer.

### Future Perspective

Previous study groups have proposed possible explanations for the association between meningioma and breast cancer, such as hormone receptor expression in both diseases or adverse effects of radiation therapy and hormonal therapy. Moreover, the role of overactivation of the *MYC* oncogene has been hypothesized.^18,38^

Both types of tumors have been linked to hormone receptors in their genesis and progression. Approximately 88% of meningiomas express progesterone receptors (PR), but only 30% express estrogen receptors (ER).^58,59^ The ER is expressed in 70% of breast cancers; the PR is expressed in 50% of ER-positive breast cancers but is rarely seen in ER-negative breast cancers.^60,61,62,63^ However, the studies by Criscitiello et al^10^ and Lopez-Rivera et al^8^ did not find any significant association between the hormonal receptor status of breast cancer and the risk of meningioma.

Ionizing radiation is a well-known risk factor for meningioma and is frequently used as a treatment for breast cancer.^64,65,66^ This might be a contributing factor to the cooccurrence of both diseases. However, the study by Lopez-Rivera et al^8^ failed to show a difference in the observed risk of meningioma among patients treated with vs without radiotherapy for breast cancer. Hormone therapy, particularly synthetic progestagens, has been associated with an increased risk of both meningioma and breast cancer.^58,67,68^ Undoubtedly, further research is required to unravel the potential risk factors related to the cooccurrence of benign meningioma and malignant breast cancer, and the findings from this meta-analysis should be considered for implementation in guidelines regarding screening for these 2 diseases in female patients, specifically screening for breast cancer in female patients with meningioma.

### Limitations

This study has several limitations. All previous studies have expressed the occurrence of both diseases in terms of incidence per year. Incidence rates are more widely used than rates of prevalence to express the prevalence of a disease. Incidence baseline values in the population per year and country are widely available. However, performing a meta-analysis on the basis of incidence rates was not possible, as we did not have information in all studies about which diagnosis was made first and at what point in the study the diagnoses were established; therefore, we could not correct for the number of study years. For these reasons, we opted to use prevalence. At the end of the study, the total number of individuals with both meningioma and breast cancer in the total population was assessed. These prevalences were compared with baseline prevalences. However, data on prevalence were scarce. We used a baseline prevalence measured in Belgium in 2020. For breast cancer, we also used prevalence rates from 2020, but we had sufficient breast cancer data to adjust the values for each country where the study was performed. Older, reliable prevalence rates were not available in the literature. As the rates of incidence of both meningiomas and breast cancer are increasing with time, we most likely overestimated them in our meta-analyses; this fact further strengthens our conclusions. Among studies using baseline prevalence of meningiomas, 45% date from before 2000. Baseline prevalence was based on histologically confirmed meningiomas, and in some of the studies, meningioma was diagnosed based on radiological findings without histologic confirmation.

On the other hand, the prevalence of breast cancer in the global population used for our study was overestimated, since 2020 numbers were used and adjusted per country, 54% date from before 2000. Since the incidence of breast cancer is increasing each year, the study was carried out under the least favorable conditions, and it must be concluded that the OR and relative risk are potentially greater than those estimated in our work. On the other hand, we were unable to adjust for age, race, and diagnosis year when calculating the OR and relative risk due to a lack of data.

A limitation of our study could be linked to a possible publication bias in the meta-analysis of group 1. Another limitation of the study is that we were unable to extract sufficient data from the investigated studies regarding the first diagnosed tumor, histology, hormonal receptor expression, treatment received, age at diagnosis of the first tumor, and time between diagnoses of the 2 diseases to conduct subanalyses.

In addition, we would also like to report that 1 ad hoc exclusion criteria was used due to a reporting bias in 1 study (Maiuri et al^43^), discovered later in the analysis process. This also constitutes a limitation of the study.

## Conclusions

To our knowledge, this study is the largest systematic review and the first meta-analysis on the association between meningioma and breast cancer, and we found that female patients diagnosed with a meningioma had nearly 10-fold higher odds of breast cancer compared with the general female population. Based on this finding, female patients with meningioma should be screened more intensively for breast cancer.

## Notes

Supplement.
Data Sharing Statement

## References

[1] Ostrom QT, Cioffi G, Waite K, Kruchko C, Barnholtz-Sloan JS. CBTRUS statistical report: primary brain and other central nervous system tumors diagnosed in the United States in 2014-2018. Neuro Oncol. 2021;23(12)(suppl 2):iii1-iii105. doi:10.1093/neuonc/noab200 34608945 PMC8491279

[2] Bondy M, Ligon BL. Epidemiology and etiology of intracranial meningiomas: a review. J Neurooncol. 1996;29(3):197-205. doi:10.1007/BF00165649 8858525

[3] Low JT, Ostrom QT, Cioffi G, . Primary brain and other central nervous system tumors in the United States (2014-2018): a summary of the CBTRUS statistical report for clinicians. Neurooncol Pract. 2022;9(3):165-182. doi:10.1093/nop/npac015 35601966 PMC9113389

[4] Goldbrunner R, Stavrinou P, Jenkinson MD, . EANO guideline on the diagnosis and management of meningiomas. Neuro Oncol. 2021;23(11):1821-1834. doi:10.1093/neuonc/noab150 34181733 PMC8563316

[5] World Health Organization. Estimated cumulative risk of incidence in 2020, World, females, all ages (excl. NMSC). Accessed May 16, 2023. https://gco.iarc.fr/today/online-analysis-multi-bars?v=2020&mode=cancer&mode_population=countries&population=900&populations=900&key=cum_risk&sex=2&cancer=39&type=0&statistic=5&prevalence=0&population_group=0&ages_group%5B%5D=0&ages_group%5B%5D=17&nb_item

[6] Lapresle J, Netsky MG, Zimmerman HM. The pathology of meningiomas; a study of 121 cases. Am J Pathol. 1952;28(5):757-791. 12976523 PMC1937375

[7] Schoenberg BS, Christine BW, Whisnant JP. Nervous system neoplasms and primary malignancies of other sites: the unique association between meningiomas and breast cancer. Neurology. 1975;25(8):705-712. doi:10.1212/WNL.25.8.705 1171403

[8] Lopez-Rivera V, Zhu P, Dono A, . Increased risk of subsequent meningioma among women with malignant breast cancer. World Neurosurg. 2020;139:e271-e285. doi:10.1016/j.wneu.2020.03.203 32298823

[9] Ji J, Sundquist J, Sundquist K. Association of tamoxifen with meningioma: a population-based study in Sweden. Eur J Cancer Prev. 2016;25(1):29-33. doi:10.1097/CEJ.0000000000000133 25642792 PMC4885544

[10] Criscitiello C, Disalvatore D, Santangelo M, . No link between breast cancer and meningioma: results from a large monoinstitutional retrospective analysis. Cancer Epidemiol Biomarkers Prev. 2014;23(1):215-217. doi:10.1158/1055-9965.EPI-13-1041 24165579

[11] Abrahão-Machado LF, Abrahao-Machado EF, Abrahao-Machado ECF, . Tumor-to-tumor metastasis: intracranial meningioma harboring metastatic breast carcinoma. Ann Clin Pathol. 2015;3(2):1049. doi:10.47739/2373-9282/1049

[12] Aghi M, Kiehl TR, Brisman JL. Breast adenocarcinoma metastatic to epidural cervical spine meningioma: case report and review of the literature. J Neurooncol. 2005;75(2):149-155. doi:10.1007/s11060-005-1408-4 16132512

[13] Anlyan FH, Heinzen BR, Carras R. Metastasis of tumor to second different tumor: collision tumors. JAMA. 1970;212(12):2124. doi:10.1001/jama.1970.03170250078030 5467793

[14] Başaran AE, Kiesel B, Frischer JM, . Intrameningioma metastasis: a wolf in sheep’s clothing—experience from a series of 7 cases. World Neurosurg. 2019;132:169-172. doi:10.1016/j.wneu.2019.08.097 31491578

[15] Cervoni L, Salvati M, Gagliardi D, Delfini R. Metastasis of breast carcinoma to intracranial meningioma. Case report. Neurosurg Rev. 1994;17(3):233-236. doi:10.1007/BF00418442 7838405

[16] Chou LW, Ho KH, Fong CM. Intracranial meningioma with metastatic breast carcinoma. Ann Oncol. 1992;3(5):409-410. doi:10.1016/S0923-7534(19)65250-6 1616896

[17] Doron Y, Gruszkiewicz J. Metastasis of invasive carcinoma of the breast to an extradural meningioma of the cranial vault. Cancer. 1987;60(5):1081-1084. doi:10.1002/1097-0142(19870901)60:5<1081::AID-CNCR2820600526>3.0.CO;2-N 3300947

[18] Elmaci L, Ekinci G, Kurtkaya O, Sav A, Pamir MN. Tumor in tumor: metastasis of breast carcinoma to intracranial meningioma. Tumori. 2001;87(6):423-427. doi:10.1177/030089160108700613 11989598

[19] Haar F, Patterson RH Jr. Surgical for metastatic intracranial neoplasm. Cancer. 1972;30(5):1241-1245. doi:10.1002/1097-0142(197211)30:5<1241::AID-CNCR2820300515>3.0.CO;2-5 4117255

[20] Hockley AD. Metastatic carcinoma in a spinal meningioma. J Neurol Neurosurg Psychiatry. 1975;38(7):695-697. doi:10.1136/jnnp.38.7.695 1159441 PMC1083249

[21] Joglekar VM, Davis CH, Blakeney CG. Metastasis of carcinoma to meningioma and glioma. Acta Neurochir (Wien). 1981;58(1-2):67-74. doi:10.1007/BF01401684 7282462

[22] Jun P, Garcia J, Tihan T, McDermott MW, Cha S. Perfusion MR imaging of an intracranial collision tumor confirmed by image-guided biopsy. AJNR Am J Neuroradiol. 2006;27(1):94-97. 16418364 PMC7976103

[23] Kepes JJ. Cellular whorls in brain tumors other than meningiomas. Cancer. 1976;37(5):2232-2237. doi:10.1002/1097-0142(197605)37:5<2232::AID-CNCR2820370512>3.0.CO;2-Y 177186

[24] Lanotte M, Benech F, Panciani PP, Cassoni P, Ducati A. Systemic cancer metastasis in a meningioma: report of two cases and review of the literature. Clin Neurol Neurosurg. 2009;111(1):87-93. doi:10.1016/j.clineuro.2008.07.011 18930586

[25] Lee A, Wallace C, Rewcastle B, Sutherland G. Metastases to meningioma. AJNR Am J Neuroradiol. 1998;19(6):1120-1122. 9672023 PMC8338649

[26] Lin JW, Su FW, Wang HC, . Breast carcinoma metastasis to intracranial meningioma. J Clin Neurosci. 2009;16(12):1636-1639. doi:10.1016/j.jocn.2009.02.020 19766009

[27] Lodrini S, Savoiardo M. Metastases of carcinoma to intracranial meningioma: report of two cases and review of the literature. Cancer. 1981;48(12):2668-2673. doi:10.1002/1097-0142(19811215)48:12<2668::AID-CNCR2820481219>3.0.CO;2-N 7306923

[28] Magdelenat H, Pertuiset BF, Poisson M, Philippon J. Steroid receptor status difference in recurrent intracranial meningioma and breast cancer in the same patient. J Neurooncol. 1986;4(2):155-157. doi:10.1007/BF00165376 3023558

[29] Markopoulos C, Sampalis F, Givalos N, Gogas H. Association of breast cancer with meningioma. Eur J Surg Oncol. 1998;24(4):332-334. doi:10.1016/S0748-7983(98)80019-X 9725004

[30] Miller RE. Breast cancer and meningioma. J Surg Oncol. 1986;31(3):182-183. doi:10.1002/jso.2930310309 3724170

[31] Pöyhönen L, Heikkinen J, Vehkalahti I. Two different primary tumours of the brain in a patient with breast cancer. Eur J Nucl Med. 1979;4(6):483-484. doi:10.1007/BF00300851 230047

[32] Savoiardo M, Lodrini S. Hypodense area within a meningioma: metastasis from breast cancer. Neuroradiology. 1980;20(2):107-110. doi:10.1007/BF00339557 7422122

[33] Schmitt HP. Metastases of malignant neoplasms to intracranial tumours: the “tumour-in-a-tumour” phenomenon. Virchows Arch A Pathol Anat Histopathol. 1984;405(1):155-160. doi:10.1007/BF00694933 6438898

[34] Theologides A, Lee J-C. Tumor to tumor metastasis. JAMA. 1972;219(3):384. doi:10.1001/jama.1972.03190290070023 5066633

[35] Watanabe T, Fujisawa H, Hasegawa M, . Metastasis of breast cancer to intracranial meningioma: case report. Am J Clin Oncol. 2002;25(4):414-417. doi:10.1097/00000421-200208000-00019 12151976

[36] Zon LI, Johns WD, Stomper PC, . Breast carcinoma metastatic to a meningioma: case report and review of the literature. Arch Intern Med. 1989;149(4):959-962. doi:10.1001/archinte.1989.00390040151036 2650648

[37] Burns PE, Jha N, Bain GO. Association of breast cancer with meningioma: a report of five cases. Cancer. 1986;58(7):1537-1539. doi:10.1002/1097-0142(19861001)58:7<1537::AID-CNCR2820580726>3.0.CO;2-P 3742472

[38] Caroli E, Salvati M, Giangaspero F, Ferrante L, Santoro A. Intrameningioma metastasis as first clinical manifestation of occult primary breast carcinoma. Neurosurg Rev. 2006;29(1):49-54. doi:10.1007/s10143-005-0395-4 16133455

[39] Bonito D, Giarelli L, Falconieri G, Bonifacio-Gori D, Tomasic G, Vielh P. Association of breast cancer and meningioma: report of 12 new cases and review of the literature. Pathol Res Pract. 1993;189(4):399-404. doi:10.1016/S0344-0338(11)80326-2 8351240

[40] Knuckey NW, Stoll J Jr, Epstein MH. Intracranial and spinal meningiomas in patients with breast carcinoma: case reports. Neurosurgery. 1989;25(1):112-116. doi:10.1227/00006123-198907000-00022 2666877

[41] Kubo M, Fukutomi T, Akashi-Tanaka S, Hasegawa T. Association of breast cancer with meningioma: report of a case and review of the literature. Jpn J Clin Oncol. 2001;31(10):510-513. doi:10.1093/jjco/hye109 11696622

[42] Lieu AS, Hwang SL, Howng SL. Intracranial meningioma and breast cancer. J Clin Neurosci. 2003;10(5):553-556. doi:10.1016/S0967-5868(02)00305-3 12948458

[43] Maiuri F, Cappabianca P, Iaconetta G, D’Acunzi G. Meningiomas associated with brain metastases. Zentralbl Neurochir. 2002;63(3):111-115. doi:10.1055/s-2002-35823 12457336

[44] Mehta D, Khatib R, Patel S. Carcinoma of the breast and meningioma: association and management. Cancer. 1983;51(10):1937-1940. doi:10.1002/1097-0142(19830515)51:10<1937::AID-CNCR2820511031>3.0.CO;2-F 6831359

[45] Salvati M, Cervoni L. Association of breast carcinoma and meningioma: report of nine new cases and review of the literature. Tumori. 1996;82(5):491-493. doi:10.1177/030089169608200517 9063531

[46] Smith FP, Slavik M, MacDonald JS. Association of breast cancer with meningioma: report of two cases and review of the literature. Cancer. 1978;42(4):1992-1994. doi:10.1002/1097-0142(197810)42:4<1992::AID-CNCR2820420445>3.0.CO;2-O 213189

[47] Smith-Behn J. Relationship between breast cancer and meningioma. South Med J. 1992;85(2):146-147. doi:10.1097/00007611-199202000-00007 1310817

[48] Wang D, Sadek AR, Vaseeharan S, Manivannan S, Walker M, Nader-Sepahi A. Presentation and management of spinal meningioma and its association with breast carcinoma-case series and systematic review. Br J Neurosurg. Published online April 18, 2022. doi:10.1080/02688697.2022.2061419 35435093

[49] Adami HO, Bergkvist L, Krusemo U, Persson I. Breast cancer as a risk factor for other primary malignant diseases: a nationwide cohort study. J Natl Cancer Inst. 1984;73(5):1049-1055. doi:10.1093/jnci/73.5.1049 6593483

[50] Custer BS, Koepsell TD, Mueller BA. The association between breast carcinoma and meningioma in women. Cancer. 2002;94(6):1626-1635. doi:10.1002/cncr.10410 11920521

[51] Helseth A, Mørk SJ, Glattre E. Neoplasms of the central nervous system in Norway—V: meningioma and cancer of other sites: an analysis of the occurrence of multiple primary neoplasms in meningioma patients in Norway from 1955 through 1986. APMIS. 1989;97(8):738-744. doi:10.1111/j.1699-0463.1989.tb00471.x 2765276

[52] Jacobs DH, McFarlane MJ, Holmes FF. Female patients with meningioma of the sphenoid ridge and additional primary neoplasms of the breast and genital tract. Cancer. 1987;60(12):3080-3082. doi:10.1002/1097-0142(19871215)60:12<3080::AID-CNCR2820601236>3.0.CO;2-X 3677029

[53] Jacobs DH, Holmes FF, McFarlane MJ. Meningiomas are not significantly associated with breast cancer. Arch Neurol. 1992;49(7):753-756. doi:10.1001/archneur.1992.00530310101020 1497504

[54] Milano MT, Grossman CE. Meningioma in breast cancer patients: population-based analysis of clinicopathologic characteristics. Am J Clin Oncol Cancer Clin Trials. 2014;40(1):11-16. doi:10.1097/COC.0000000000000052 24577166

[55] Rao G, Giordano SH, Liu J, McCutcheon IE. The association of breast cancer and meningioma in men and women. Neurosurgery. 2009;65(3):483-489. doi:10.1227/01.NEU.0000350876.91495.E0 19687693

[56] Malmer B, Tavelin B, Henriksson R, Grönberg H. Primary brain tumours as second primary: a novel association between meningioma and colorectal cancer. Int J Cancer. 2000;85(1):78-81. doi:10.1002/(SICI)1097-0215(20000101)85:1<78::AID-IJC14>3.0.CO;2-S 10585587

[57] Ben Lassan M, Laitman Y, Keinan-Boker L, Silverman B, Friedman E. Secondary cancer after meningioma diagnosis: an Israeli national study. Cancer Causes Control. 2022;33(10):1277-1284. doi:10.1007/s10552-022-01609-3 35871439

[58] Champeaux-Depond C, Weller J, Froelich S, Sartor A. Cyproterone acetate and meningioma: a nationwide-wide population based study. J Neurooncol. 2021;151(2):331-338. doi:10.1007/s11060-020-03672-9 33394263

[59] Hage M, Plesa O, Lemaire I, Raffin Sanson ML. Estrogen and progesterone therapy and meningiomas. Endocrinology. 2022;163(2):1-10. doi:10.1210/endocr/bqab259 34935947

[60] Scabia V, Ayyanan A, De Martino F, . Estrogen receptor positive breast cancers have patient specific hormone sensitivities and rely on progesterone receptor. Nat Commun. 2022;13(1):3127. doi:10.1038/s41467-022-30898-0 35668111 PMC9170711

[61] Grann VR, Troxel AB, Zojwalla NJ, Jacobson JS, Hershman D, Neugut AI. Hormone receptor status and survival in a population-based cohort of patients with breast carcinoma. Cancer. 2005;103(11):2241-2251. doi:10.1002/cncr.21030 15844176

[62] Patani N, Martin LA, Dowsett M. Biomarkers for the clinical management of breast cancer: international perspective. Int J Cancer. 2013;133(1):1-13. doi:10.1002/ijc.27997 23280579

[63] Leong ASY, Zhuang Z. The changing role of pathology in breast cancer diagnosis and treatment. Pathobiology. 2011;78(2):99-114. doi:10.1159/000292644 21677473 PMC3128144

[64] Brenner AV, Sugiyama H, Preston DL, . Radiation risk of central nervous system tumors in the Life Span Study of atomic bomb survivors, 1958-2009. Eur J Epidemiol. 2020;35(6):591-600. doi:10.1007/s10654-019-00599-y 31982981 PMC7329623

[65] Preston DL, Ron E, Yonehara S, . Tumors of the nervous system and pituitary gland associated with atomic bomb radiation exposure. J Natl Cancer Inst. 2002;94(20):1555-1563. doi:10.1093/jnci/94.20.1555 12381708

[66] National Cancer Registry and Analysis Service. Chemotherapy, Radiotherapy and Surgical Tumour Resections in England: 2013-2020 diagnoses (April 2023). Accessed May 22, 2023. http://www.ncin.org.uk/cancer_type_and_topic_specific_work/topic_specific_work/main_cancer_treatments

[67] Abenhaim HA, Suissa S, Azoulay L, Spence AR, Czuzoj-Shulman N, Tulandi T. Menopausal hormone therapy formulation and breast cancer risk. Obstet Gynecol. 2022;139(6):1103-1110. doi:10.1097/AOG.0000000000004723 35675607

[68] Hoisnard L, Laanani M, Passeri T, . Risk of intracranial meningioma with three potent progestogens: a population-based case-control study. Eur J Neurol. 2022;29(9):2801-2809. doi:10.1111/ene.15423 35621369 PMC9543130

