# Supplementary material for: The Association Between Meningioma and Breast Cancer: A Systematic Review and Meta-analysis
Source: JAMA Netw Open. 2023 Jun 16;6(6):e2318620. doi: 10.1001/jamanetworkopen.2023.18620 (PMC10276307; doi:10.1001/jamanetworkopen.2023.18620)
Supplement: Supplement. — Data Sharing Statement [file jamanetwopen-e2318620-s001.pdf]

# Data Sharing Statement

Degeneffe. The Association Between Meningioma and Breast Cancer. *JAMA Netw Open*. Published June 16, 2023. doi:10.1001/jamanetworkopen.2023.18620

**Data**
**Data available:** No
